# Stable fitness during COVID-19: Results of serial testing in a cohort of youth with heart disease

**DOI:** 10.3389/fped.2023.1088972

**Published:** 2023-02-20

**Authors:** Adam W. Powell, Wayne A. Mays, Samuel G. Wittekind, Clifford Chin, Sandra K. Knecht, Sean M. Lang, Alexander R. Opotowsky

**Affiliations:** ^1^ Department of Pediatrics, University of Cincinnati College of Medicine, Cincinnati, OH, United States; ^2^ The Heart Institute, Cincinnati Children’s Hospital Medical Center, Cincinnati, OH, United States

**Keywords:** COVID-19, exercise changes, body composition, cardiopulmonary exercise test (CPET), congenital heart disaese

## Abstract

**Background:**

Little is known about how sport and school restrictions early during the novel coronavirus 2019 (COVID-19) pandemic impacted exercise performance and body composition in youth with heart disease (HD).

**Methods:**

A retrospective chart review was performed on all patients with HD who had serial exercise testing and body composition *via* bioimpedance analysis performed within 12 months before and during the COVID-19 pandemic. Formal activity restriction was noted as present or absent. Analysis was performed with a paired *t*-test.

**Results:**

There were 33 patients (mean age 15.3 ± 3.4 years; 46% male) with serial testing completed (18 electrophysiologic diagnosis, 15 congenital HD). There was an increase in skeletal muscle mass (SMM) (24.1 ± 9.2–25.9 ± 9.1 kg, *p* < 0.0001), weight (58.7 ± 21.5–63.9 ± 22 kg, *p* < 0.0001), and body fat percentage (22.7 ± 9.4–24.7 ± 10.4%, *p* = 0.04). The results were similar when stratified by age <18 years old (*n* = 27) or by sex (male 16, female 17), consistent with typical pubertal changes in this predominantly adolescent population. Absolute peak VO_2_ increased, but this was due to somatic growth and aging as evidenced by no change in % of predicted peak VO_2_. There remained no difference in predicted peak VO_2_ when excluding patients with pre-existing activity restrictions (*n* = 12). Review of similar serial testing in 65 patients in the 3 years before the pandemic demonstrated equivalent findings.

**Conclusions:**

The COVID-19 pandemic and related lifestyle changes do not appear to have had substantial negative impacts on aerobic fitness or body composition in children and young adults with HD.

## 1. Introduction

Concerns about cardiac damage following coronavirus disease 2019 (COVID-19) and society-wide mitigation strategies disrupted children's lives during the COVID-19 pandemic, particularly during the early stages. Policies, such as school and sport restriction, dramatically altered daily routines, leading to more school-based and leisure screen time and less time in active play with their peers (1). Decreased physical activity because of COVID-19 related restrictions has been shown in multiple studies in pediatrics (1–3). Little is known about the effect this has had on fitness and body composition in pediatric and congenital heart disease (CHD), individuals who are often already deconditioned. The only known pediatric CHD study included a combination of individuals with and without heart disease, reporting a decline in peak oxygen consumption (VO_2_) on serial exercise testing performed during the pandemic (4).The aims of this study were to: (1) understand how COVID-19 mitigation strategies affected fitness and also body composition in youth with heart disease, and (2) determine if children with heart disease and pre-existing exercise restrictions were more likely to have alterations in their fitness or body composition compared to without such restriction.

## 2. Materials and methods

This study was approved by the Cincinnati Children's Hospital Medical Center Institutional Review Board, and informed consent was not required for this study.

### 2.1. Patients

We identified all individuals with heart disease ≤21 years old at the time of the first test, and who underwent a routine cardiopulmonary exercise test (CPET) at Cincinnati Children's Hospital within a year before and after March 21, 2020 (i.e., the date that extensive COVID-19 mitigation strategies including school and sports cancellations were established locally). This group we defined as the “COVID lockdown” study group. Heart disease was defined as either electrocardiographic (i.e., Long QT syndrome, catecholaminergic polymorphic ventricular tachycardia) or CHD (bicuspid aortic valve, Fontan physiology, etc). We included only those for whom exercise testing was performed as routine standard of care and not secondary to cardiac symptoms or concern for deterioration (i.e., testing to assess for adequate beta-blockade if long QT patients, evaluate for serial changes in peak VO_2_ in CHD patients for prognostic purposes, ST segment/T wave changes in patients with left ventricular outflow tract obstruction). This was evaluated on chart review utilizing the electronic medical record. Additional exclusion criteria included a submaximal test (details below) and missing data. Additionally, to determine if any changes in fitness were secondary to COVID-19 mitigation or rather typical fitness changes in patients with heart disease, an age, sex, and diagnosis-matched historical control group was performed for those with serial CPET performed during the 3 years prior to the COVID-19 pandemic. To help account for other confounders, following initial result reporting a sensitivity analysis was performed to evaluate for differences in serial testing by excluding patients with pre-existing activity restrictions, adult patients, and those with a percent predicted peak VO_2_ < 80%. Lastly, all patients tested during the COVID-19 pandemic required a negative PCR test on a nasopharyngeal swab prior to their CPET.

### 2.2. Study measures

#### 2.2.1. Baseline data

We extracted demographic and other baseline data from the electronic medical record including age, sex, height, weight, race, medications, cardiac pacemaker, and documented sport restrictions.

#### 2.2.2. Bioimpedance assessment

Anthropometric data were measured by Bioimpedance assessment (BIA) (InBody370; InBody, Cerritos, CA, United States) immediately before the CPET, as previously described (5). Bioelectrical impedance analysis (BIA) is part of our local standard of care prior to CPET and is an alternative approach to measure body composition with good agreement to dual energy x-ray absorptiometry (6, 7).

#### 2.2.3. CPET

Exercise testing was performed on a stationary cycle ergometer (Corival; Lode; Groningen, The Netherlands) with an individualized incremental ramp protocol. The rate of increase was chosen by experienced clinical exercise physiologists based on the patient's body size and expected fitness, targeting an exercise duration of approximately 10 min. Cardiopulmonary responses to exercise were assessed breath-by-breath (Ultima CardiO2; MGC Diagnostics; Saint Paul, MN, United States). Criteria for a maximal effort exercise test were that 2 of the following 3 criteria were met: respiratory exchange ratio >1.10; maximal heart rate greater >85% of the age-predicted maximum (220 - age in years); or maximal Rating of Perceived Exertion ≥18 on a 6–20 scale (8). Predicted peak VO_2_ was calculated per Wasserman et al. and Cooper et al. (9, 10). In adult patients with a body mass index (BMI) <18 and >25, the underweight and overweight regression equations were used, respectively (9).

### 2.3. Statistics

Descriptive normally distributed data are presented as mean ± standard deviation. Differences between serial tests for both the COVID-19 lockdown and historical control groups were assessed using a paired *t*-test. To determine if there was a difference in the baseline fitness testing of both the control and COVID groups, a 2-sided *t*-test was performed with each group's initial CPET results. All presented *p*-values are two-tailed (where applicable) and differences and associations were considered significant when *p* < 0.05. Statistical analyses were performed using JMP®, Version 15 (SAS Institute Inc., Cary, NC).

## 3. Results

There were a total of 48 patients who underwent serial testing, with the first test performed within 12 months before and the second test performed within 12 months after the COVID-19 lockdown. There were 15 patients excluded (6 tested because of concern for clinical decompensation, 6 submaximal effort tests, 3 missing data). There were 33 remaining patients (mean age 15.3 ± 3.4 years at the time of the first test; 46% male) who were included in the analysis. Underlying cardiac diagnoses are presented in Table 1. Primary diagnosis was related to electrophysiologic disease in 18 patients, while 15 had structural heart disease. Of note, 36% (12/33) were restricted from sports before COVID-19 with the reason for sports restriction being concern about ventricular arrhythmia in 10 patients and severe left ventricular outflow tract obstruction in 2 patients. In the COVID group, 58% (19/33) were prescribed beta-blockers. None had a cardiac pacemaker, had medication changes, or experienced unexpected arrhythmias during the test.

**Table 1:** Demographic data for the young cardiology patients with serial cardiopulmonary exercise tests in the 12 months before and after the COVID-19 lockdown.

Overall sample size	33
Sex	Male 16
	Female 17
Race	Caucasian 29
	African-American 2
	Asian 2
Activity restriction	Yes 12
	No 21
Beta-blocker medication	Yes 19
	No 14
Primary diagnosis	Long QT syndrome 15
	Fontan circulation 4
	Cardiomyopathy 4
	CPVT 2
	Double outlet right ventricle 2
	Bicuspid aortic valve 2
	D-TGA 1
	PA/IVS 1
	SVT with aborted cardiac arrest 1
	Genotype positive, phenotype negative cardiomyopathy 1

CPVT, catecholaminergic polymorphic ventricular tachycardia; D-TGA, complete transposition of the great arteries; PA/IVS, pulmonary atresia with intact ventricular septum; SVT, supraventricular tachycardia.

There was no significant difference in sport restriction and beta-blocker usage between the post COVID-19 lockdown and historical control groups. No patients were diagnosed with COVID-19 prior to their exercise test.

In the COVID-19 lockdown group, the mean time between tests was 1.1 years, ranging from 0.6 to 1.5 years. There was an increase in weight (58.7 ± 21.5–63.9 ± 22 kg, *p* < 0.0001), skeletal muscle mass (24.1 ± 9.2–25.9 ± 9.1 kg, *p* < 0.0001), and body fat percentage (22.7 ± 9.4–24.7 ± 10.4%, *p* = 0.04) without significant change in height (165.5 ± 16.4–167 cm ± 14.2 cm, *p* = 0.1) or body mass index (21.7 ± 4.8 vs. 22.5 ± 5.5 kg/m^2^, *p* = 0.2) (Table 2).

**Table 2:** Results of serial testing of anthropometric data as measured by bioimpedance analysis and cardiopulmonary exercise testing in 33 pediatric and congenital heart disease patients before and during the COVID-19 lockdown.

	Test 1	Test 2	*p*-value
Age (years)	15.3 ± 3.4	16.4 ± 3.3	—
Height (cm)	165.5 ± 16.4	167 ± 14.2	0.13
Weight (kg)	58.7 ± 21.5	63.9 ± 22.0	<0.0001
BMI (kg/m^2^)	21.7 ± 4.8	22.5 ± 5.5	0.17
Skeletal muscle mass (kg)	24.1 ± 9.2	25.9 ± 9.1	<0.0001
Body fat (%)	22.7 ± 9.4	24.7 ± 10.4	0.04
Respiratory exchange ratio	1.2 ± 0.1	1.3 ± 0.1	0.09
Work (watts)	155.1 ± 67.6	155.1 ± 63.9	0.9
Peak HR (% predicted)	79.7 ± 11.9	76.8 ± 12.6	0.08
Peak VO_2_ (ml/min)	1,833 ± 785	1,958 ± 836	0.03
Peak VO_2_ (ml/kg/min)	31.2 ± 7.5	31.1 ± 8.9	0.9
Percent predicted peak VO_2_ (%)	78.6 ± 13.1	79.3 ± 16.9	0.8
Ventilatory anaerobic threshold (%)	54.9 ± 12.9	52.1 ± 12.9	0.4
Peak systolic blood pressure (mmHg)	160.2 ± 26.3	159.9 ± 23.9	0.9
Peak SpO_2_ (%)	99.7 ± 0.8	99.5 ± 1.0	0.2
VE/VCO_2_ slope	31.7 ± 4.9	30.5 ± 7.5	0.3

Data are presented as mean ± SD. A paired *t*-test was performed to determine differences between paired data. A *p*-value < 0.05 was considered significant.

Cm, centimeters; kg, kilogram; m, meters; BMI, body mass index; HR, heart rate; VO_2_, oxygen consumption; SpO_2_, oxygen saturation measured *via* pulse oximeter; VE/VCO_2_ slope, minute ventilation/carbon dioxide production slope.

There was an increase in absolute peak VO_2_ (1,832.9 ± 784.8–1,958.2 ± 835.9 ml/min, *p* = 0.03), but no change in peak VO_2_ when expressed as a percent of predicted values or when indexed to body mass (78.6 ± 13.1–79.3 ± 16.9%, *p* = 0.8; 31.2 ± 7.5–31.1 ± 8.9 ml/kg/min, *p* = 0.9). There was no significant change between tests in any of the other exercise variables (Table 2).

When further evaluating the COVID-19 lockdown group, body composition and CPET differences by sex are provided in Table 3. Of note, male patients had an increase in weight (68.9 ± 24–74.6 ± 24.2 kg, *p* = 0.02) and skeletal muscle mass (29.5 ± 9.1–32.7 ± 8.3 kg, *p* = 0.002) without a significant change in adiposity (18.9 ± 10–18.8 ± 9.5% body fat, *p* = 0.8) or height (176.1 ± 12.2–176.4 cm ± 11.9 cm, *p* = 0.9). Female patients had an increase in height (155.4 ± 13.3–158.2 cm ± 10.2 cm, *p* = 0.008), weight (49 ± 13.253.8 ± 14 kg, *p* < 0.0001), skeletal muscle mass (18.7 ± 5.4–19.8 ± 4.2 kg, *p* = 0.005), and body fat (26.4 ± 7.3–29.8 ± 8.4%, *p* = 0.03). Male patients had a significant difference between absolute peak VO_2_ but not peak VO_2_ indexed to weight or as a percent of predicted values. There were no other significant differences based on sex.

**Table 3:** Differences in anthropometric and exercise testing data by sex in the pediatric and congenital heart disease patients tested before and during the COVID-19 lockdown.

	Male (*n* = 16)				Female (*n* = 17)	
Test 1	Test 2	*p*-value		Test 1	Test 2	*p*-value
16.5 ± 3.4	17.6 ± 3.3	—	Age (years)	14.2 ± 3.0	15.3 ± 3.0	—
176.1 ± 12.2	176.4 ± 11.9	0.9	Height (cm)	155.4 ± 13.3	158.2 ± 10.2	0.008
68.9 ± 24.1	74.6 ± 24.2	0.02	Weight (kg)	49 ± 13.2	53.8 ± 14.0	<0.001
22.9 ± 6.0	23.1 ± 6.1	0.8	BMI (kg/m^2^)	20.5 ± 3.1	22 ± 4.9	0.2
29.5 ± 9.1	32.7 ± 8.3	0.002	SMM (kg)	18.7 ± 5.4	19.8 ± 4.2	0.005
18.9 ± 10.0	18.8 ± 9.5	0.8	Body fat (%)	26.4 ± 7.3	29.8 ± 8.4	0.03
1.3 ± 0.10	1.3 ± 0.10	0.3	RER	1.2 ± 0.08	1.3 ± 0.10	0.2
202.7 ± 65.8	201.7 ± 60.9	0.8	Work (watts)	110.2 ± 26.2	111.2 ± 21.2	0.8
81.4 ± 11.8	75.9 ± 14.3	0.08	Peak HR (% pred)	78 ± 12.2	77.6 ± 11.2	0.7
2,375 ± 757	2,567 ± 768	0.03	Peak VO_2_ (ml/min)	1,323 ± 354	1,385 ± 352	0.4
35.3 ± 8.5	36.0 ± 8.2	0.6	Peak VO_2_ (ml/kg/min)	27.3 ± 3.5	26.4 ± 6.8	0.6
79.4 ± 16.3	81.4 ± 15.5	0.4	Peak VO_2_ (% pred)	77.8 ± 9.7	77.4 ± 18.4	0.9
55.1 ± 15.7	51.5 ± 12.9	0.4	VAT (%)	54.8 ± 10.6	52.8 ± 13.3	0.8
170.0 ± 26.7	169.4 ± 25.8	0.9	Peak SBP (mmHg)	151.1 ± 23.0	150.9 ± 18.5	0.9
99.8 ± 0.5	99.9 ± 0.3	0.4	Peak SpO_2_ (%)	99.7 ± 0.9	99.1 ± 1.3	0.05
31.2 ± 4.3	28.4 ± 5.6	0.02	VE/VCO_2_ slope	32.0 ± 5.4	32.3 ± 8.5	0.7

Data are presented as mean ± SD. A paired *t*-test was performed to determine differences between paired data. A *p*-value < 0.05 was considered significant.

cm, centimeters; kg, kilogram; m, meters; BMI, body mass index; SMM, skeletal muscle mass; HR, heart rate; VO_2_, oxygen consumption; % pred, percent predicted; VAT, ventilatory anaerobic threshold; SBP, systolic blood pressure; SpO_2_, oxygen saturation measured *via* pulse oximeter; VE/VCO_2_ slope, minute ventilation/carbon dioxide production slope.

When comparing with the cohort that had testing in the years prior to the COVID-19 pandemic (historical control) (*N* = 65; mean age 14.9 ± 3.3 years at the first test; 49% male), there was no difference in the sex, diagnoses, age, or size between groups. On comparing baseline CPET results between the COVID-19 lockdown and historical control groups, there were no significant differences in any of the CPET parameters studied. In the historical control group, there was no significant change in absolute peak VO_2_ (1,826.8 ± 696.8–1,866.7 ± 655.6 ml/min, *p* = 0.4) or percent predicted peak VO_2_ (78.3 ± 15.3–75.9 ± 17.7%, *p* = 0.2), though there was a decrease in peak heart rate (82.1 ± 11.4–78.1 ± 14.0%, *p* < 0.001). The ventilatory anaerobic threshold was lower on serial testing in the cohort with testing before the pandemic (54.3 ± 13.2–50.9 ± 14.6, *p* = 0.02) (Supplementary Table S1).

### 3.1. Sensitivity analysis

Excluding the 12 patients with pre-existing activity restrictions, the remaining 21 patients in the COVID lockdown group also experienced no change in percent predicted peak VO_2_. There was no significant change in the percent predicted peak VO_2_ between tests, although the absolute values trended towards abnormal in both the activity restricted (77.2 ± 8.3–76.8 ± 15.1%, *p* = 0.9) and not restricted (79.4 ± 15.4–80.8 ± 18.1%, *p* = 0.7) groups. Skeletal muscle mass increased in both those who did and did not have an activity restriction, but those who were restricted had a smaller increase (restricted 24.7 ± 7.9–26.5 ± 7.4 kg, *p* = 0.02; not restricted 23.7 ± 10.1–26.3 ± 10.2 kg, *p* = 0.0006). Neither group had a significant increase in body fat percentage >2% (restricted 24.3 ± 7.9–26.3 ± 9.3%, *p* = 0.3; not restricted 21.7 ± 10.3–23.7 ± 11.1%, *p* = 0.07).

Results were similar after excluding the 7 patients in the COVID lockdown group who were >18 years old at the initial test (Supplementary Table S2). The 7 patients >18 years old had a significant decrease in total work (221.4 ± 47.1–207.3 ± 49.4 watts, *p* = 0.04), but there was no change in other CPET variables. When removing patients with a peak VO_2_ < 80%, there remained no difference in percent predicted peak VO_2_ in those with more normal fitness (89.6 ± 11.2 vs. 89.3 ± 16.4, *p* = 0.9). Lastly, the groups with a primary electrophysiologic diagnosis were also similar to those with a structural heart disease diagnosis in terms of change in percent predicted peak VO_2_.

## 4. Discussion

Findings from this observational study revealed no significant change in the aerobic capacity or body composition, beyond those associated with puberty, from before to after the COVID-19 pandemic and related societal lockdown. This is notable, as there has previously been concern that COVID mitigation could affect exercise habits in this population (1, 11). There were no differences in fitness when comparing serial testing separated by either sex, activity restriction, or in the matched paired historical control group. There were changes in body composition seen between tests, but this was likely secondary to normal pubertal maturation in a largely pediatric cohort.

There have been reports of decreased physical activity related to COVID-19 mitigation strategies both in healthy children and in those with heart disease (1, 11). Whether or not our cohort engaged in less physical activity is unknown, but their objective measures of fitness did not change, which is the opposite of what was reported by Burstein DS et al. (4). This may mean many of the patients were already deconditioned at the time of the initial CPET, as evidenced by an average peak VO_2_ of ∼78% of predicted, compared to ∼96% in the Burstein DS et al. cohort (4). Additionally, the lack of change in fitness may be secondary to pre-existing activity restrictions applied by the patients' medical teams, although the lack of change in VO_2_ in those not activity restricted argues against this. Lastly, this may mean that the introduction of lockdown measures did not alter their physical activity as they were already sedentary and deconditioned, which is not unusual for a typical congenital heart disease cohort. Prior to COVID-19, youth with congenital heart disease were shown to be more sedentary than their peers (12, 13). Thus, the introduction of lockdown measures may not have resulted in a significant change in their physical activity levels. This is further supported by the lack of change in the already low peak VO_2_ on serial testing in the pre-COVID control group.

This study continues to highlight the need to encourage physical activity in youth with congenital heart disease. While the absence of direct negative effects from the lockdown on fitness in children with heart disease is reassuring, this population, both in our cohort and others, has been shown to be significantly deconditioned (14–16). This is meaningful, since decreased peak VO_2_ has both negative prognostic and functional implications (17–19). Recreational and competitive activities should be encouraged in this population with an eye towards the risk: benefit calculation, balancing the concerns for sudden cardiac arrest during athletics, and the long-term morbidity and mortality that arises from sedentary behaviors. For individuals so severely deconditioned that participation in organized sports would be impractical, exercise prescription and structured cardiac rehabilitation should be encouraged to improve cardiopulmonary fitness (20).

While this study focused on evaluating the effect of COVID-19 mitigation strategies on the body composition of children and young adults with heart disease, body composition changed during the study period in a pattern that is likely consistent with normal pubertal changes (21). Increase in both skeletal muscle mass and body fat percentage results in an increase in total weight in a pubertal population, which was seen in our cohort. The increase in the total weight for the males was largely driven by increases in skeletal muscle mass while the increase in weight for the females was largely due to increased adiposity; this is in keeping with typical sex-specific pubertal body composition changes (21). These changes remained when the 7 patients older than 18 years-old were removed from the analysis, again supporting expected somatic growth as the likely explanation. The lack of significant change in predicted peak VO_2_ in this cohort further supports that these body composition changes were most likely related to normal, expected growth. Unfortunately, secondary to the retrospective design of this study we do not have specific information on the exact pubertal stage of these patients. This should be taken into account for future pediatric studies involving serial body composition measurements.

There were other limitations to this analysis. The available sample size limits statistical power, and a subset of the null findings may represent false negative findings, particularly those with a small effect size. The comparison group lacked body composition data, as the InBody Bioimpedance scale was not widely used in our laboratory until early 2019; thus it is unknown if the changes in body composition in the study cohort is reproduced in the age, sex, and diagnosis-matched cohort. Additionally, questions on specific exercise habits and sports participation are not routinely asked by all of the providers in our hospital. Additional sampling bias may also exist secondary to the retrospective design of the study.

## 5. Conclusion

The COVID-19 pandemic and related lifestyle changes do not appear to have had an substantial negative impact on aerobic fitness in children and young adults with heart disease. While this is largely reassuring, these finding may be in part related to pre-existing chronic deconditioning, pre-limited participation in organized sport, or sampling bias.

## Data Availability

The raw data supporting the conclusions of this article will be made available by the authors, without undue reservation.
