# Supplementary material for: Stable fitness during COVID-19: Results of serial testing in a cohort of youth with heart disease
Source: Front Pediatr. 2023 Feb 20;11:1088972. doi: 10.3389/fped.2023.1088972 (PMC9986439; doi:10.3389/fped.2023.1088972)
Supplement: Supplementary file 1 [file Table1.docx]

**Supplemental Table 1**: Results of serial cardiopulmonary exercise testing in pediatric and congenital heart disease patients (N=65; male 49%) performed within the three years prior to the COVID-19 pandemic.

|  | **Test 1** | **Test 2** | **P-value** |
| --- | --- | --- | --- |
| **Age (years)** | 14.9±3.3 | 16.1±3.3 | --- |
| **Height (cm)** | 161.7±16.1 | 164.4±14.4 | <0.0001 |
| **Weight (kg)** | 60.4±21.7 | 66.4±23.7 | <0.0001 |
| **BMI (kg/m^2^)** | 22.6±6 | 23.9±6.6 | <0.0001 |
| **Respiratory exchange ratio** | 1.2±0.09 | 1.2±0.1 | 0.003 |
| **Work (watts)** | 145.1±57.4 | 153.9±58.0 | 0.0002 |
| **Peak HR (% predicted)** | 82.1±11.4 | 78.1±14.0 | 0.0007 |
| **Peak VO_2_ (ml/min)** | 1826.8±696.8 | 1866.7±655.6 | 0.37 |
| **Peak VO_2_ (ml/kg/min)** | 32.2±7.3 | 30.1±10.6 | 0.3 |
| **Peak VO_2_ (% predicted)** | 78.3±15.3 | 75.9±17.7 | 0.2 |
| **Ventilatory anaerobic threshold (%)** | 54.3±13.2 | 50.9±14.6 | 0.02 |
| **Peak systolic blood pressure (mmHg)** | 161.2±32.1 | 180.9±161.9 | 0.3 |
| **Peak SpO_2_ (%)** | 96.9±3.6 | 96.8±3.7 | 0.5 |
| **VE/VCO_2_ slope** | 32.2±6.2 | 32.2±7.5 | 0.9 |

Data are presented as mean±SD. A paired t-test was performed to determine differences between paired data. A p value <0.05 was considered significant

Abbreviations: cm (centimeters), kg (kilogram), m (meters), BMI (body mass index), HR (heart rate), VO_2_ (oxygen consumption), VAT (ventilatory anaerobic threshold), SBP (systolic blood pressure), SpO_2_ (oxygen saturation measured via pulse oximeter), VE/VCO_2_ slope (minute ventilation/carbon dioxide production slope).
