# Supplementary material for: Stable fitness during COVID-19: Results of serial testing in a cohort of youth with heart disease
Source: Front Pediatr. 2023 Feb 20;11:1088972. doi: 10.3389/fped.2023.1088972 (PMC9986439; doi:10.3389/fped.2023.1088972)
Supplement: Supplementary file 2 [file Table2.docx]

**Supplemental Table 2**: Comparison of adult (18 years of age and older) and pediatric patients

|  | **Pediatric (n=26)** |  |  |  | **Adult**  **(n=7)** |  |
| --- | --- | --- | --- | --- | --- | --- |
| **Test 1** | **Test 2** | **P-value** |  | **Test 1** | **Test 2** | **P-value** |
| 13.6±2.6 | 14.7±2.6 | --- | **Age (years)** | 18.8±1.3 | 19.9±1.3 | --- |
| 161±15.4 | 162.9±12.8 | 0.2 | **Height (cm)** | 181.8±6.6 | 182.4±6.8 | 0.07 |
| 52.8±16.2 | 57.1±15.6 | <0.001 | **Weight (kg)** | 80.5±25.7 | 89.1±25.1 | 0.1 |
| 20.9±3.7 | 21.6±4.4 | 0.4 | **BMI (kg/m^2^)** | 24.3±7.5 | 25.8±7.8 | 0.2 |
| 21.1±7.4 | 22.9±7.3 | <0.001 | **SMM (kg)** | 34.2±7.4 | 36.3±7.3 | 0.06 |
| 23.8±8.9 | 25.3±10.4 | 0.2 | **Body fat (%)** | 19.0±10.8 | 22.4±10.9 | 0.08 |
| 1.2±0.09 | 1.3±0.1 | 0.09 | **RER** | 1.3±0.1 | 1.3±0.2 | 0.6 |
| 137.2±61.3 | 141.1±60.5 | 0.1 | **Work (watts)** | 221.4±47.1 | 207.3±49.4 | 0.04 |
| 82.0±11.2 | 78.8±12.5 | 0.1 | **Peak HR (% predicted)** | 71.1±11.2 | 69.4±11.2 | 0.6 |
| 1641.0±712.4 | 1757.2±748.1 | 0.06 | **Peak VO_2_ (ml/min)** | 2546.0±645.6 | 274.9±754.3 | 0.3 |
| 30.8±8.0 | 30.6±9.2 | 0.8 | **Peak VO_2_ (ml/kg/min)** | 32.4±5.5 | 32.9±7.9 | 0.8 |
| 77.8±14.5 | 77.8±18.2 | 0.9 | **Peak VO_2_ (%)** | 81.4±5.3 | 84.9±10.3 | 0.3 |
| 55.2±14 | 53.4±13.7 | 0.6 | **VAT (%)** | 53.7±7.5 | 47.7±9.1 | 0.4 |
| 157.6±28 | 158±24.6 | 0.9 | **Peak SBP (mmHg)** | 170±17.1 | 166.9±21.1 | 0.7 |
| 99.7±0.9 | 99.5±1.1 | 0.3 | **Peak SpO_2_ (%)** | 100±0 | 99.7±0.5 | 0.2 |
| 32.2±4.8 | 31.9±7.7 | 0.9 | **VE/VCO_2_ slope** | 29.5±5 | 25.7±3.7 | 0.03 |

Data are presented as mean±SD. A paired t-test was performed to determine differences between paired data. A p value <0.05 was considered significant

Abbreviations: cm (centimeters), kg (kilogram), m (meters), BMI (body mass index), SMM (skeletal muscle mass), HR (heart rate), VO_2_ (oxygen consumption), VAT (ventilatory anaerobic threshold), SBP (systolic blood pressure), SpO_2_ (oxygen saturation measured via pulse oximeter), VE/VCO_2_ slope (minute ventilation/carbon dioxide production slope).
